# Advice on describing Bayesian analysis of neutron and X-ray reflectometry^1^

**DOI:** 10.1107/S1600576722011426

**Published:** 2023-02-01

**Authors:** Andrew R. McCluskey, Andrew J. Caruana, Christy J. Kinane, Alexander J. Armstrong, Thomas Arnold, Joshaniel F. K. Cooper, David L. Cortie, Arwel V. Hughes, Jean-Francois Moulin, Andrew R. J. Nelson, Wojciech Potrzebowski, Vladimir Starostin

**Affiliations:** a European Spallation Source ERIC, PO Box 176, Lund, SE-22100, Sweden; b ISIS Neutron and Muon Source, Rutherford Appleton Laboratory, Didcot, Oxfordshire OX11 0QX, United Kingdom; c Australian Nuclear Science and Technology Organisation, Lucas Heights, New South Wales, Australia; d German Engineering Material Science at Heinz Maier-Leibnitz Zentrum, Helmholtz-Zentrum Hereon, Lichtenbergstraße 1, 85748 Garching, Germany; e Institute of Applied Physics, University of Tübingen, Auf der Morgenstelle 10, 72076 Tübingen, Germany; DESY, Hamburg, Germany

**Keywords:** reflectometry, reflectivity, Bayesian analysis, FAIR data standards

## Abstract

As a result of the availability of modern software and hardware, Bayesian analysis is becoming more popular in neutron and X-ray reflectometry analysis. The understandability and replicability of these analyses may be harmed by inconsistencies in how the probability distributions central to Bayesian methods are represented in the literature. Herein advice is provided on how to report the results of Bayesian analysis as applied to neutron and X-ray reflectometry. This includes the clear reporting of initial starting conditions, the prior probabilities, the results of any analysis and the posterior probabilities that are the Bayesian equivalent of the error bar, to enable replicability and improve understanding. It is believed that this advice, grounded in the authors’ experience working in the field, will enable greater analytical reproducibility in the work of the reflectometry community, and improve the quality and usability of results.

## Abstract 2

The members of the Open Reflectometry Standards Organisation outline their best practice for reporting the results of Bayesian analysis of reflectometry measurements. Following this advice will enable greater reproducibility and improve understanding.

## 1. Introduction

Neutron and X-ray reflectometry are powerful tools to probe the interfacial structure of materials (Lovell & Richardson, 1999 ▸). However, as a result of the ‘phase problem’ the analysis of these techniques is ill-posed in nature, as there are multiple possible solutions (Majkrzak & Berk, 1995 ▸). This has led to the use of Bayesian analysis, where some prior understanding of the system is used to aid our understanding of a reflectivity profile (Sivia *et al.*, 1991 ▸; Geoghegan *et al.*, 1996 ▸; Sivia & Webster, 1998 ▸). Recently, developments in the availability of computer software for reflectometry analysis that includes Bayesian functionality, such as *Refl1D* (Kienzle *et al.*, 2021*b* ▸), *refnx* (Nelson & Prescott, 2019 ▸), *anaklasis* (Koutsioubas, 2021 ▸) and *RasCAL* (Hughes, 2021 ▸), which implement sampling methods from *bumps* (Kienzle *et al.*, 2021*a* ▸), *emcee* (Foreman-Mackey *et al.*, 2019 ▸) and *dynesty* (Speagle, 2020 ▸), have led to an increase in the utilization of Bayesian methods by the reflectometry community (McCluskey *et al.*, 2019 ▸, 2020 ▸).

This article will focus on the best practice for reporting the results from Bayesian and sampling-based analysis of neutron and X-ray reflectivity data. This work will not introduce Bayesian or sampling methods for neutron and X-ray reflectometry analysis. For those unfamiliar with these techniques, we suggest the work of Sivia and co-workers (Sivia & Webster, 1998 ▸; Sivia & Skelling, 2006 ▸) and more recent work focusing on reflectometry analysis (Hughes *et al.*, 2019 ▸; McCluskey *et al.*, 2020 ▸; Nelson & Prescott, 2019 ▸; Aboljadayel *et al.*, 2021 ▸). We hope that this paper will inform best practices in data sharing from reflectometry analysis and inspire software developers to enable these to be accessed easily by the user.

Reflectometry analysis can be described, in the most simplistic terms, as a comparison and refinement of a model based on some parameters **x** to reproduce a reflectivity data set **D**. This refinement process involves comparing the model and the data and calculating some goodness-of-fit value or likelihood *p*(**D**|**x**), and modifying the model to optimize the goodness of fit or maximize the likelihood. A commonly used goodness-of-fit parameter is the χ^2^ parameter which is found as (Nelson & Prescott, 2019 ▸)

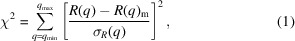

where *R*(*q*) and *R*(*q*)_m_ are, respectively, the measured and modelled reflectivity at a given *q*, while σ_*R*_(*q*) is the uncertainty associated with the measured reflectivity at each *q*. Here, *q* = (4π/λ)sinθ is the measured momentum transfer, where θ is half the scattering angle and λ is the wavelength of the incident radiation. Under an assumption of normally distributed residuals 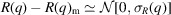 , the likelihood is related to the χ^2^ variable in the following way: 

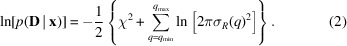

The input for this refinement process is the model and some initial parameter values, each of which may be an absolute value or a parameter range, depending on the refinement algorithm. The output is a set of values for **x**, potentially with associated error bars – when these are present they typically describe a standard deviation from the mean of a Gaussian probability distribution. For Bayesian sampling processes, the input is a probability distribution for each parameter, known as the prior. The sampling process gives a probability distribution, the posterior, that defines the relative likelihood of different values of each parameter, and from this we can report statistical measures, *e.g.* mode/mean/median. This process implicitly assumes that the data are completely reduced, all experimental parameters are accounted for, uncertainties are accurately described and the model can accurately describe the data.

The input required depends on a minimization algorithm being used, with some algorithms requiring a single starting guess (such as traditional Newtonian methods) and others taking a range of potential values (more common in stochastic approaches like differential evolution). The nature of these inputs defines the results of the analysis, and therefore it is of the utmost importance that these are communicated as part of a publication describing the work. Furthermore, the minimization is often performed with bounds in place, defining that the parameter values will lie within a given range. This range can be thought of as having a prior probability distribution *p*(**x**), where values of **x** outside of this range have a probability of 0. Even when a non-Bayesian approach is used in the analysis (*i.e.* Bayes’ theorem is not utilized), the result where bounds are set would be analogous to a Bayesian analysis with a uniform prior probability.

The optimized parameters from the minimization algorithm, which depend on the particular algorithm used, often include some statistical uncertainty. This uncertainty comes from an assumption of normally distributed parameters (Bevington & Robinson, 2002 ▸), but Bayesian sampling approaches make no assumption of an underlying statistical distribution. How these statistical uncertainties are found is beyond the scope of this work, but it is important to acknowledge that this uncertainty typically assumes that the probability distribution of the parameter is Gaussian in nature. This probability distribution is either the partial likelihood or posterior, the latter when some prior is included and Bayes’ theorem is applied. The posterior describes our understanding of the parameter values given the data that were measured. When Bayesian modelling is used and the prior is included, the posterior probability is found as 

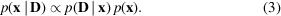

Therefore, when Bayesian modelling is performed, the priors and likelihood are of fundamental importance to the results that are obtained (the posterior) and any scientific conclusions that are drawn. We note that equation (3) omits the normalization term, the Bayesian evidence [*p*(**D**)], which is discussed in detail elsewhere (Sivia & Webster, 1998 ▸; McCluskey *et al.*, 2020 ▸) and can be omitted when model comparison is not being performed.

The use of Bayesian inference can be valuable in the interpretation of reflectivity data, but inconsistency in the description of the process will result in an analysis that cannot be reproduced or easily understood. This can range from not reporting the priors applied to each parameter (*e.g.* the lower/upper limits for a uniform distribution that applies box bounds) to failing to describe the complete sampling chain of a Markov chain Monte Carlo sampling, or details of any autocorrelation analysis (the last of which the authors of this work admit to being guilty of; McCluskey *et al.*, 2019 ▸). In this article, we outline the best practice for reporting the results of Bayesian analysis for neutron and X-ray reflectometry, and we hope that this work will engage others to consider carefully how they report this information. Furthermore, uptake of the approaches discussed herein will lead to greater clarity about the models and assumptions used in, and the reproducibility of, our analyses.

## 2. Prior

The most common probability distributions that are used for a prior are uniform between a lower and upper bound or over a half-closed interval, where only a lower or upper bound is defined. The use of a bounded parameter along with some traditional χ^2^ minimization method and a parameter with a uniform prior and a Bayesian maximum *a posteriori* approach will lead to the same result. For priors that are uniform it is important that the upper and lower bounds are reported, and this can be achieved with a simple table (see Table 1 ▸ for an example) to be included in the article or supplementary information. Note that this table also gives information on ‘constrained’ values, where in some analyses the parameters are not allowed to vary; these constrained values can have a significant impact on the result of any analysis and therefore must also be given.

Currently, the use of non-uniform informative priors is less common in reflectometry analysis. However, the increasing popularity of Bayesian methods and interest in using complementary methods for analysis means that these are likely to become more popular in the coming years. Here we will define two potential types of informative prior probabilities, those that can be described with a mathematical function and those that cannot, for example arising from the application of a sampling-based analysis of a complementary technique.

**Figure 1:**
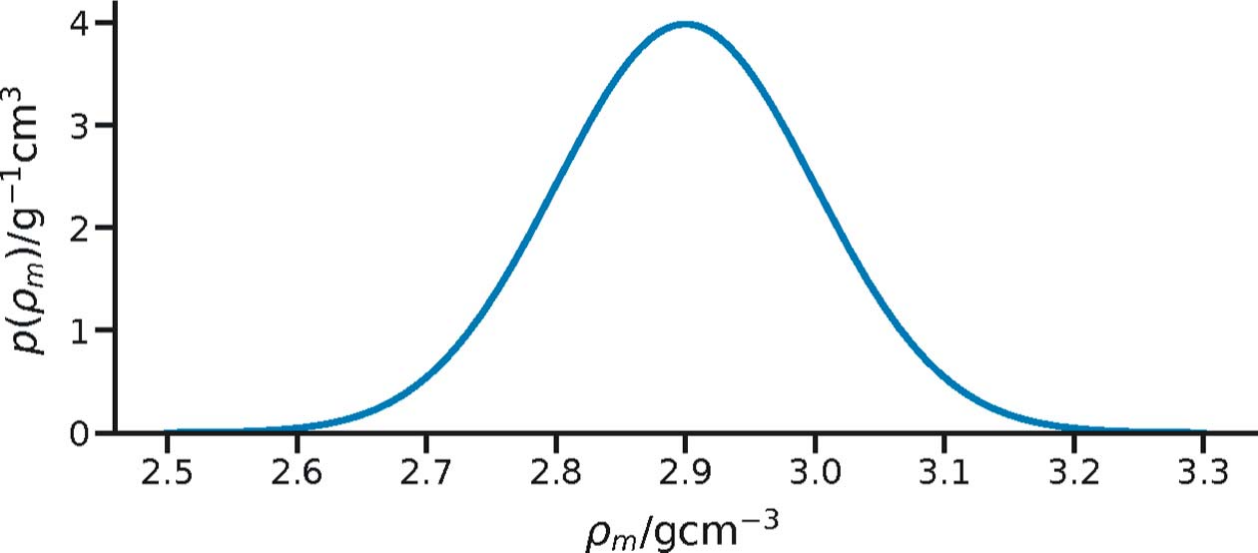
A prior probability distribution for Si_3_N_4_ with a density of ρ_m_ = 2.9 ± 0.1 g cm^−3^.

When a prior probability can be described with a mathematical function, this should be done by providing this function in the clearest possible language. For example, if the prior is taken from a single complementary measurement that is defined as a value with some uncertainty, which represents a normal distribution with a mean and standard deviation, this information should be provided. This is shown in Fig. 1 ▸ for the density of silicon nitride (Si_3_N_4_) produced by atomic layer deposition (Knoops *et al.*, 2015 ▸) which is used to inform the value of the scattering length density for some layer of the material. Such a prior probability distribution could be described in several ways: graphically (Fig. 1 ▸), in prose as being ‘normally distributed with a mean of 2.9 g cm^−3^ and a standard deviation of 0.1 g cm^−3^’, mathematically as

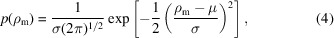

where μ = 2.9 g cm^−3^ and σ = 0.1 g cm^−3^, or more concisely as *p*(ρ_m_) ≃ 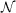 (μ = 2.9 g cm^−3^, σ = 0.1 g cm^−3^). The same descriptive approach could be taken for any common statistical distribution, including log-normal or truncated normal distributions.

**Figure 2:**
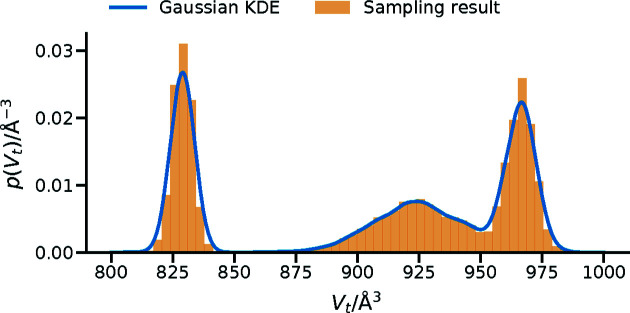
A hypothetical prior probability distribution for a dipalmitoyl phosphatidylcholine lipid that could arise from a molecular dynamics simulation (orange histogram) and a Gaussian kernel density estimation for the probability distribution using a bandwidth factor of 0.05 (blue line).

It is possible that the prior distribution cannot be described with a simple mathematical function, multi-modal priors being an example; if it is a multimodal model result from some other sampling method, then the chain from the prior sampling should be given. The chain is all of the samples investigated in the sampling and should be reported, although in the case that this chain is very large a subsampled object may be reported, in which case the autocorrelation analysis performed should be described (see Section 4 for a more complete discussion of this). To use such a prior probability in Bayesian analysis, some functional description of the prior must be generated, and most commonly this will be some kernel density estimation; when this is used it is also necessary to state the structure of the kernel being used. An example of this is shown in Fig. 2 ▸, where the prior probability for the volume of a phospholipid tail could be found from molecular dynamics simulation; there are three common conformers that the lipid is likely to have.

## 3. Likelihood

Bayes’ theorem [given in equation (3)] consists of the product of the prior and the likelihood. The former describes our current understanding of the parameters before we conduct any experiments, while the latter describes how well the data are described by the model parameters. Although equation (2) is a common approach to quantify how well the data are described, it assumes a normally distributed uncertainty for the measured reflectivity value. While a normally distributed uncertainty is the most common, it may not be accurate in all circumstances. For example when low numbers of counts are present, a Poisson uncertainty may offer a more accurate description, in which case the likelihood function could be changed to one which reflects a multi-dimensional Poisson distribution (Lass *et al.*, 2021 ▸). Additionally, the likelihood function may be modified by weighting the data at high *q*, such as by replacing *R*(*q*) in equation (1) with 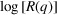 or *R*(*q*)*q* ^4^. These aspects make it very important to state explicitly how the likelihood for a given model and data is calculated (giving the analysis package used and version number, and if not the default the likelihood option).

## 4. Posterior

Bayesian analysis methods typically involve using some sampling process, such as Markov chain Monte Carlo, to estimate the posterior probability distributions for each of the parameters. Assuming there are *m* parameters under investigation, the posterior will be an *m*-dimensional probability distribution. The result of a Bayesian sampling process is a ‘chain’ consisting of *n* samples for each parameter. Therefore, the full chain has a shape (*m*, *n*). Typically these are histogrammed to show the probability of different values of the parameters. However, to identify independent (non-correlated) samples in the chain, autocorrelation analysis (Sokal, 1997 ▸) may be performed and the chain ‘thinned’. We will not cover autocorrelation analysis in detail, other than to say that it helps to identify the length of separation required for samples to be independent, and thinning means that we have only included samples separated by this length in the final chain. Additionally, it is valuable to report the use of convergence diagnostics, such as the Gelman–Rubin statistic (Gelman & Rubin, 1992 ▸), which can assist in determining if a chain appropriately describes a posterior.

**Figure 3:**
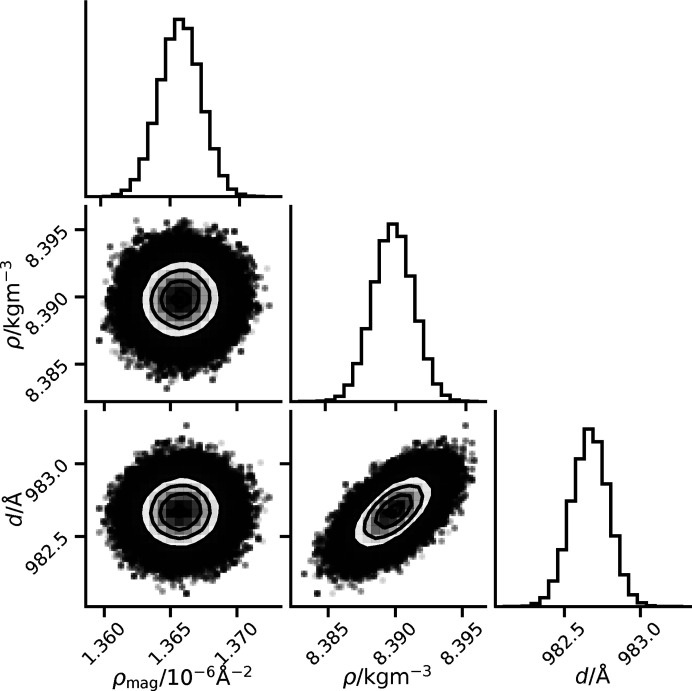
An example of a graphical depiction of the unthinned posterior as a corner plot (produced using the corner.py package; Foreman-Mackey, 2016 ▸), representing a three-dimensional probability distribution showing the posterior distribution for the parameters of nickel magnetic scattering length density, nickel mass density and nickel layer thickness, from the analysis of a nickel layer on a silicon block (Caruana & Kinane, 2022 ▸).

Either the full posterior chain or the thinned chain should be reported, along with details of any autocorrelation analysis to accompany any Bayesian or sampling analysis. This will allow the best replication and verification of any results obtained from the data. Furthermore, large output files such as these chains can be easily shared using some versioned data repository, such as Zenodo (European Organization for Nuclear Research & OpenAIRE, 2013 ▸) or those available at specific institutions. Additionally, to allow the reader to interpret the sampled posterior quickly, a graphical description (such as that in Fig. 3 ▸) should be included, at a minimum, in the supplementary information of the work. The importance of presenting the full posterior graphically lies in the ease with which it enables interpretation of the correlations between parameters through this medium. For example, in Fig. 3 ▸ the ellipsoidal probability distribution (for the *d*/ρ parameters) indicates correlation.

To report values for parameters and some form of statistical uncertainty, two approaches can be taken from the posterior chain. The first is to use some known statistical distribution that describes the samples well. This is best defined for a normal distribution, for which there are statistical tests to check normality, such as the D’Agostino and Pearson test (D’Agstino, 1971 ▸; D’Agostino & Pearson, 1973 ▸) (which is available in the *SciPy* library as scipy.stats.normaltest; Virtanen *et al.*, 2020 ▸). As with all statistical tests, this requires some threshold value to be defined to reject the null hypothesis, and for this value we recommend 0.001 but accept that this is at the discretion of the user. If the parameter distribution passes a statistical test for a given distribution type, this can be quoted in the report, with information about the distribution type and threshold value used, and the distribution can be described on the basis of fitted parameters of the distribution as discussed above for the Gaussian distribution. For example, the three parameters in Fig. 3 ▸ pass this statistical test, with *p* values greater than 0.01 when 1000 random samples are used, and therefore we can quote the parameters as normal distributions: ρ_mag_ = (1.366 ± 0.001) × 10^−6^ Å^−2^, ρ_m_ = (8.390 ± 0.001) kg m^−3^ and *d* = (982.668 ± 0.121) Å.

If it is not possible to describe the *m*-dimensional distribution using some statistical test and a common distribution type, then confidence intervals can be given. Where these are used the percentage of the confidence interval must be defined alongside each. In addition to these confidence intervals, it is typically most accurate to give the maximum probability value for the parameter, rather than the numerical mean which may sit in a region of low probability. When reporting these quantiles of interest, we should assess how much precision we ascribe to them, which is typically achieved by defining some Monte Carlo standard error (MCSE) (Vehtari *et al.*, 2021 ▸). This is the variability that would be observed should the sampling process be repeated. There are a range of approaches to computing the MCSE, including the mcse method from the *ArviZ* package (Kumar *et al.*, 2019 ▸). It is important to check that the MCSE is small enough to report the level of precision desired for a given parameter.

Regardless of how the chain is communicated, as components of a fully reproducible analysis the author should also give details of the software packages, scripts and data used to produce the analysis, and any random number seeds that were defined. This means that if the chain is not available, the reader can rerun the sampling and replicate the results. Included in this is information regarding specific version numbers for different software packages, as these can create irreplicable results between version numbers. We emphasize the value of openly reporting the posteriors of some Bayesian sampling approaches. All posteriors may be utilized as prior probabilities in subsequent analyses; therefore, by sharing posteriors we enable improved analysis in future.

## 5. Conclusions

The use of Bayesian analysis in neutron and X-ray reflectometry is increasing and, alongside this, there is a need for analytical clarity and reproducibility. We have outlined the best practice, based on experience, for reporting information from Bayesian analysis. Specifically, we have outlined how the prior probabilities used to inform our analyses should be stated, as either uniform or more informed probability distributions that may or may not be described mathematically. We have mentioned the importance of including the specific likelihood function used in an analysis. Additionally, we have described how best to present the results from a Bayesian analysis in a clear and precise fashion, including the importance of reporting statistical tests and confidence intervals.

We hope that this advice will be taken on by the reflectometry community, and that in future there will be greater consistency and clarity in the reporting of results from Bayesian methods. Furthermore, we hope that developers of analysis software will take this work as a call to arms to include these best practices as easy-to-access methods in their software. Finally, if the results of neutron and X-ray Bayesian analysis are reported as outlined in this work, then the analysis will be both reproducible and comprehensible.

## 6. Data availability

Supporting information is available as follows. All analysis or plotting scripts and data files for this work, allowing for a fully reproducible and automated analysis workflow using *showyourwork!* (Luger, 2022 ▸; Luger *et al.*, 2021 ▸), are available at https://github.com/arm61/reporting_sampling (https://doi.org/10.5281/zenodo.6874559) under an MIT licence, while the paper is shared under a CC BY-SA 4.0 licence (McCluskey *et al.*, 2022 ▸). The data shown in Fig. 3 ▸ are also available under a CC BY-SA 4.0 licence (Caruana & Kinane, 2022 ▸).

## Figures and Tables

**Table 1:** An example of the presentation of uniform priors in a tabular format Reproduced from McCluskey *et al.* (2020 ▸), where each parameter was either constrained to a given value or sampled within the prior range.

Parameter	Constrained value	Prior range
*d*_h_ (Å)	10.0	[8.0, 16.0)
*V*_h_ (Å^3^)	339.5	[300.0, 380.0)
*d*_t_ (Å)	21.0	[10.0, 26.0)
ϕ_t_	1.0	[0.5, 1.0]
*V*_t_ (Å^3^)	850.4	[800.0, 1000.0)
σ (Å)	2.9	[2.9, ∞)
